# Effect of methylphenidate on functional controllability: a preliminary study in medication-naïve children with ADHD

**DOI:** 10.1038/s41398-022-02283-4

**Published:** 2022-12-17

**Authors:** Teague R. Henry, Nicholas D. Fogleman, Tehila Nugiel, Jessica R. Cohen

**Affiliations:** 1 Department of Psychology and School of Data Science, University of Virginia, Charlottesville, VA USA; 2 Department of Psychiatry, University of North Carolina at Chapel Hill, Chapel Hill, NC USA; 3 Department of Psychology and Neuroscience, University of North Carolina at Chapel Hill, Chapel Hill, NC USA; 4 Carolina Institute for Developmental Disabilities, University of North Carolina at Chapel Hill, Chapel Hill, NC USA; 5 Biomedical Research Imaging Center, University of North Carolina at Chapel Hill, Chapel Hill, NC USA

**Keywords:** ADHD, Molecular neuroscience

## Abstract

Methylphenidate (MPH) is the recommended first-line treatment for attention-deficit/hyperactivity disorder (ADHD). While MPH’s mechanism of action as a dopamine and noradrenaline transporter blocker is well known, how this translates to ADHD-related symptom mitigation is still unclear. As functional connectivity is reliably altered in ADHD, with recent literature indicating dysfunctional connectivity dynamics as well, one possible mechanism is through altering brain network dynamics. In a double-blind, placebo-controlled MPH crossover trial, 19 medication-naïve children with ADHD underwent two functional MRI scanning sessions (one on MPH and one on placebo) that included a resting state scan and two inhibitory control tasks; 27 typically developing (TD) children completed the same protocol without medication. Network control theory, which quantifies how brain activity reacts to system inputs based on underlying connectivity, was used to assess differences in average and modal functional controllability during rest and both tasks between TD children and children with ADHD (on and off MPH) and between children with ADHD on and off MPH. Children with ADHD on placebo exhibited higher average controllability and lower modal controllability of attention, reward, and somatomotor networks than TD children. Children with ADHD on MPH were statistically indistinguishable from TD children on almost all controllability metrics. These findings suggest that MPH may stabilize functional network dynamics in children with ADHD, both reducing reactivity of brain organization and making it easier to achieve brain states necessary for cognitively demanding tasks.

## Introduction

Neurobiologically, it has been proposed that attention-deficit/hyperactivity disorder (ADHD) emerges from disruptions to cognitive control and motivation systems [1, 2], both of which are linked to dopamine signaling [3, 4]. Not surprisingly, the recommended first-line treatment for ADHD is methylphenidate (MPH), a dopamine and noradrenaline transporter blocker [5, 6]. MPH has been shown to modulate the functional organization of the brain in both clinical and non-clinical populations [7–10]. MPH administration in children with ADHD alters functional connectivity (FC) across multiple contexts (i.e., the resting state and cognitive tasks) so that FC becomes more similar to that of typically developing (TD) children. For example, at rest MPH reduces hyperconnectivity between the default mode, executive control, and visual networks [11], as well as between cognitive control-related and reward processing-related regions [12]. During sustained attention MPH decreases fronto-striatal hyperconnectivity [13], while during working memory MPH decreases hyperconnectivity in the fronto-parietal and auditory networks [14]. Other evidence, however, points to MPH-induced changes in FC that diverge from the FC of children and adults without ADHD [15]. Further, there is currently no convergence on systems consistently impacted by MPH administration [16], as changes in FC after MPH administration in ADHD are widespread and vary as a result of specific cognitive demands. Together, this research suggests that changes in FC due to MPH are likely not localized to individual networks or connections and underscores the need for further research. Given recent literature demonstrating alterations in dynamic FC in ADHD [17–20], it is possible that an approach that examines FC from a dynamical system perspective may be better suited to identify reliable changes in FC due to MPH.

Network control theory (NCT) provides such an approach. NCT is a mathematical framework that provides tools to connect the topology of a network with the underlying dynamics of the complex system that the network describes [21, 22]. Controllability metrics operationalize the contributions of specific regions of interest (ROIs) to whole-brain dynamics. Recent studies have applied NCT to structural, white matter connectomes and have found that controllability metrics predict individual differences in cognitive performance across adolescence [23], and that the controllability of specific brain regions is related to their susceptibility to transcranial magnetic stimulation [24]. While NCT applied to structural connectomes could provide insight into a tendency to respond to a change in cognitive context (i.e., cognitive tasks) or the administration of drug, due to the physical and slow-changing nature of white matter tracts it is not able to examine how network dynamics change in response to varying cognitive contexts or single doses of MPH. To do both, it is necessary to extend NCT for use with FC networks.

Thus, in a preliminary study, we used NCT to assess how the controllability of the functional connectome differs between medication-naïve children with ADHD and TD children across cognitive contexts, and how those differences change after MPH administration in children with ADHD. This study seeks to extend prior literature using static FC approaches in a single context (i.e., rest or a cognitive task) to assess differences in children with ADHD and TD children, as well as the effects of MPH, on the functional dynamics of the whole brain across three cognitive contexts (rest and two response inhibition tasks).

Recent reviews suggest that functional connectivity within and between the default mode, fronto-parietal, salience, and attention networks is altered in children with ADHD, however the direction and magnitude of these alterations are inconsistent between studies [25, 26]. A recent meta-analysis of FC in children with ADHD found evidence for hyperconnectivity between the default mode network, fronto-parietal network, and an affective network, as well as hypoconnectivity between the fronto-parietal network and both attention and salience networks [27–29]. Given that FC alterations related to default mode, fronto-parietal, attention, and salience networks are most consistent, we hypothesized that children with ADHD would exhibit altered controllability in the same networks. Due to literature identifying disruption in motivation-related networks [25, 26, 30], we additionally hypothesized that children with ADHD would exhibit altered controllability in a reward network. Further, as task difficulty tends to modulate observed differences in FC and activation [31–35], we hypothesized that the difference in controllability would be greater during our two tasks relative to the resting state. As the direction of altered FC is inconsistent in previous literature and the relation between controllability and connectivity is complex, we did not have directional hypotheses. We developed the above hypotheses regarding which networks would be involved based on previous FC research that did not use an NCT approach. As NCT as applied to functional networks is a novel method, we took the strategy of conducting exploratory analyses that included all networks, rather than limiting our analyses to the hypothesized networks. Finally, due to the literature showing that MPH administration normalizes both functional activation and connectivity [16, 36, 37], we hypothesized that MPH administration would result in no significant differences in controllability during both rest and tasks between children with ADHD and TD children.

## Methods and materials

### Participants

Thirty-seven medication-naïve children with ADHD (M age = 9.72, SD = 1.17, 18 female) and 32 TD children (M age = 10.26, SD = 1.53, 15 female) between the ages 8 and 12 years participated in this study. Children with ADHD participated in a randomized, double-blind, placebo-controlled crossover trial with MPH, while TD children completed two identical sessions without any drug or placebo administration. Of these participants, a total of 14 children with ADHD were excluded for the following reasons: (1) insufficient scan quality (*n* = 12; see *MRI data acquisition and processing* section below for details), (2) inability to swallow the MPH/placebo pills (*n* = 1), and (3) diagnosis of autism spectrum disorder (*n* = 1). Five TD children were excluded for insufficient scan quality. Therefore, 23 medication-naïve children with ADHD (M age = 10.00, SD = 1.20, 11 female) and 27 TD children (M age = 10.38, SD = 1.45, 11 female) were included in these analyses. Children with ADHD and TD children included in analyses were not statistically different from the overall sample with regard to age, sex, race, or ADHD symptom severity. Sample characteristics are provided in Table 1. For detailed study inclusion and exclusion criteria, see Supplemental Information.

**Table 1:** Sample characteristics.

	ADHD (*n* = 23)	TD (*n* = 27)	*p*-Value^†^
Age (years)	10.00 (1.20)	10.38 (1.45)	0.32
Female	11 (47.8%)	11 (40.7%)	0.62
Estimated IQ^a^	114.13 (11.81)	116.85 (12.02)	0.43
Race			0.85
White/Caucasian	20 (86.7%)	23 (85.1%)	
Black/African-American	1 (4.3%)	1 (3.7%)	
Asian	1 (4.3%)	2 (7.4%)	
Multiracial	1 (4.3%)	1 (3.7%)	
Ethnicity			NA
Hispanic/Latino	0 (0.0%)	0 (0.0%)	
ADHD Symptoms^b^			
Inattentive	1.81 (0.79)	0.28 (0.39)	<0.001
Hyperactive/Impulsive	1.38 (0.77)	0.13 (0.19)	<0.001

All results are presented as mean (SD) or number (%). *ADHD* attention-deficit/hyperactivity disorder. *TD* typically developing.

^a^Estimated intelligence quotient (IQ) determined using the Block Design, Matrix Reasoning, Digit Span, Coding, Vocabulary, and Figure Weights subscales of the *Wechsler Intelligence Scale for Children, Fifth Edition* (WISC-V; [70]).

^b^ADHD symptoms were assessed using the *Swanson, Nolan, and Pelham Rating Scale, Version IV* (SNAP-IV; [71]).

^**†**^*p*-values were computed using two sample *t*-tests for Age, Estimated IQ, and ADHD Symptoms, and with chi-squared tests of independence for Sex and Race.

Prior evidence suggests that ~70% of children with ADHD respond favorably to MPH [5, 38, 39], with response to MPH typically defined as a reduction in ADHD symptoms over a multi-month period of treatment [40]. Given that the current study administered a single dose of MPH to medication-naïve children with ADHD, traditional examinations of the effectiveness of MPH on ADHD symptoms were not possible. Therefore, we defined response to MPH as an *acute behavioral response*, operationalized as a reduction of in-scanner head motion (FD before notch filtering, censoring, and data processing) on MPH relative to placebo. This criterion was selected for two primary reasons. First, prior evidence demonstrates that in-scanner head motion and ADHD symptoms are significantly correlated, perhaps due to common genetic influences [41]. In support of this, single dose administrations of MPH have been shown to reduce motor system excitability in both medication-naïve children with ADHD and in healthy adults [42, 43]. Second, using in-scanner motion allowed use of a single criterion across contexts rather than using task performance-based criteria (e.g., performance on the response inhibition tasks), as there was no task performance during rest and performance was different across the tasks. We only included participants in the below analyses who demonstrated an *acute behavioral response* to MPH, given that our goal was to assess possible mechanisms underlying the behavioral effects of MPH. The participants categorized as *acute behavioral responders* were different for each scan context. Thus, this criterion was applied to each scan context separately. Table 2 contains information about included participants for each functional scan context, along with the overlap of participant subsets. Thirteen children with ADHD passed *acute behavioral response* criteria for all three scan types. When restricting analyses to this subset of participants, results were consistent with the findings reported in the main text (see Supplemental Information, Table S12, Figures S1 and S2*)*.

**Table 2:** MPH Acute Response Sample Characteristics by Scan Type.

	ADHD			TD
	Rest (*n* = 18)	Go/No-Go (*n* = 19)	Rewarded Go/No-Go (*n* = 18)	All Contexts (*n* = 27)
Age (years)	9.93 (1.19)	10.04 (1.24)	10.11 (1.21)	10.38 (1.45)
Female	10 (55.6%)	9 (47.4%)	10 (55.6%)	11 (40.7%)
Framewise Displacement (mm)^a^	0.142 (0.074)	0.150 (0.075)	0.150 (0.083)	0.146 (0.075)
Overlapping (Rest)	–	15 (78.9%)	14 (77.8%)	–
Overlapping (Go/No-Go)	15 (83.3%)	–	16 (88.9%)	–
Overlapping (Rewarded Go/No-Go)	14 (77.8%)	16 (84.2%)	–	–
ADHD Symptoms^b^				
Inattentive	1.94 (0.71)	1.77 (0.81)	1.88 (0.73)	0.28 (0.39)
Hyperactive/Impulsive	1.41 (0.74)	1.35 (0.81)	1.31 (0.72)	0.13 (0.19)

All results are presented as mean (SD) or number (%). *ADHD* attention-deficit/hyperactivity disorder. *TD* typically developing.

^a^Framewise displacement reported for participants with ADHD is after MPH administration.

^b^ADHD symptoms were assessed using the Swanson, Nolan, and Pelham Rating Scale, Version IV (SNAP-IV; 32).

Finally, we tested whether in-scanner head motion (raw FD before notch filtering, censoring, and processing) was differentially related to different ADHD symptom domains to determine whether our results were driven by a single subgroup in the children with ADHD. We found that neither absolute head motion nor the change in head motion between placebo and MPH sessions was significantly related to either symptoms of hyperactivity/impulsivity or symptoms of inattention (all *p*-values > 0.29; see Supplemental Information, Tables S2 and S3).

### Study design

All study procedures were reviewed and approved by the Institutional Review Board at the University of North Carolina at Chapel Hill. Parental informed consent and child assent was obtained for all subjects. Participants completed a baseline behavioral session followed by two neuroimaging sessions that were on average 10.94 days apart (SD = 7.97 days; range = 3–42 days). MPH or placebo was administered to the participants with ADHD in a counterbalanced order that was randomly assigned and double blinded. MPH dose was 0.3 mg/kg (rounded up to the nearest 5 mg). Given the MPH half-life [44] and peak efficacy [45], MPH (or placebo) was administered 1 h prior to the start of each MRI scan. TD children participated in two identical MRI sessions with no medication or placebo (2 out of 27 TD children had only one scan session). For participants with ADHD, brain metrics were calculated separately for MPH and placebo sessions. For TD participants, brain metrics from both sessions, when available, were used in subsequent analyses. During each MRI session, participants completed two 5-min resting state scans, two 6.3-min go/no-go scans, and four 6.1-min rewarded go/no-go scans.

### Experimental paradigms

For the resting state runs, participants saw a white crosshair on a gray background and were asked to remain awake and focused on the crosshair.

For the go/no-go task, eight sports balls were separated into six ‘go’ stimuli and two ‘no-go’ stimuli; no-go stimuli were determined randomly for each participant. During each trial one sports ball was presented for 600 ms followed by a jittered intertrial interval sampled from a uniform distribution (1250–3250 ms, M = 2316 ms, SD = 588 ms). Participants were instructed to respond on go trials by pressing a button with the index finger of their right hand, and to withhold a response on no-go trials. Each of two runs consisted of 128 trials with stimuli presented in pseudorandom order such that go trials were presented in blocks of two to four, followed by a no-go trial. The rewarded go/no-go task was identical to the go/no-go task, with the addition of rewards for performance. Participants were informed that correct go responses faster than 650 ms and correct no-go non-responses would be rewarded with one cent and five cents respectively. Feedback following each trial was presented for 600 ms, and the interval between response and feedback was jittered identically to that of the intertrial interval. There were 64 trials in each of four runs for the rewarded go/no-go task. Participants were provided the amount of money earned at the end of each session.

### MRI data acquisition and processing

Scanning was performed on a 3.0-T Siemens Prisma Scanner using a 32-channel head coil at the Biomedical Research Imaging Center at the University of Carolina at Chapel Hill. A T1-weighted multiecho MPRAGE was acquired for coregistration with fMRI images (TR: 2400 ms, TE: 2.22 ms, flip angle: 8°, field of view: 256s × 256 mm, 208 slices, in-plane voxel size: 0.8 mm). BOLD signal during functional runs was acquired using a gradient-echo T2*-weighted EPI sequence (39 slices, TR: 2000 ms, TE: 25 ms, flip angle: 77°, echo spacing: 0.54 ms, field of view: 230 × 230 mm, voxel dimensions: 2.9 mm × 2.9 mm × 3 mm). Before each scan, five images were acquired and discarded to allow for magnetization to reach equilibrium. 150 volumes were acquired for each resting state scan, 195 for each go/no-go scan, and 185 for each rewarded go/no-go scan.

fMRI data preprocessing was performed using FMRIPREP [46], which includes EPI to T1w coregistration, susceptibility artifact correction, normalization to MNI space, and estimation of motion parameters. Details can be found in Supplemental Information. Following minimal preprocessing, several postprocessing steps were applied following current recommendations [47, 48]. 36-parameter nuisance regression (6 degrees of motion, global signal, white matter and CSF; with temporal, quadratic, and quadratic temporal derivatives) along with bandpass spectral filtering at 0.009–0.08 mHz was applied. Bandpass filtering was also applied to the regressor matrix [49, 50]. Censoring was performed for all timepoints with framewise displacement (FD) >0.2 mm, with five contiguous timepoints required. To identify timepoints to be censored, FD notch-filtered with a frequency band of 0.31–0.41 mHz was used instead of raw FD, given that scanner-estimated motion can be inflated due to respiration-related magnetic field disruptions [51, 52]. To combine censoring and spectral filtering, spectral interpolation based on the XCP pipeline was used [53, 54]. Postprocessing was implemented using a customized processing pipeline (clpipe) [55]. Finally, to reduce noise contamination, rest or task runs that had a mean notch-filtered FD exceeding 0.5 mm were excluded. At least 50 good timepoints for each run (minimum of 150 timepoints across runs within a task) were required to be included in analyses. The mean number of timepoints included per resting state scan (max 150) in the children with ADHD on placebo was 130.02 (range: 58–150), in the children with ADHD on MPH was 143.72 (range: 113–150) and in the TD children was 139.85 (range: 93–150). The mean number of timepoints included per regular go/no-go scan (max 195) in the children with ADHD on placebo was 158.70 (range: 71–190), in the children with ADHD on MPH was 181.13 (range: 154–191) and in the TD children was 172.12 (range: 96–195). The mean number of timepoints included per rewarded go/no-go scan (max 185) in the children with ADHD on placebo was 146.08 (range: 62–181), in the children with ADHD on MPH was 170.08 (range: 55–185) and in the TD children was 160.09 (range: 54–185). Thus, across all scan contexts the children with ADHD on placebo had the smallest number of timepoints included, the children with ADHD on MPH had the largest number of timepoints included, and the TD children had an intermediate number of timepoints included. As stated above in the *Participants* section, these criteria resulted in the exclusion of 12 children with ADHD and 5 TD children from analyses. Importantly, we conducted a set of linear regression models assessing whether children with ADHD who were included in analyses (*n* = 23) differed in terms of symptom severity (inattention or hyperactivity/impulsivity) or task performance (go/no-go or rewarded go/no-go d’) from children with ADHD who were excluded from analyses for scan quality (*n* = 12). We did not observe any significant differences (all *p*-values > 0.34; see Supplemental Information, Tables S4 and S5 for details).

### Network construction

Following processing, ROI timeseries were extracted using a whole-brain functional atlas developed by Seitzman and colleagues [56] that adds subcortical nodes to the earlier functional atlases developed by Power and colleagues [57] and Gordon and colleagues [58]. This atlas consists of 300 spherical ROIs with 4 mm radii subdivided into 13 common functional networks: the cingulo-opercular, fronto-parietal, dorsal attention, ventral attention, salience, somatomotor dorsal, somatomotor ventral, visual, auditory, medial temporal, reward, parietal memory, and default mode networks, as well as a set of unassigned ROIs. Due to limited field of view, data was not collected in all participants from portions of the cerebellum (11 ROIs), the default mode network (2 ROIs), the medial temporal network (1 ROI), and the unassigned network (2 ROIs; see Supplemental Information, Table S1 for coordinates of the missing ROIs). This resulted in a set of 284 ROIs that had sufficient data in all participants. ROI timeseries were extracted per run, detrended to remove effects of scanner drift, and standardized. Finally, ROI timeseries were concatenated across runs within task within each session.

For each task in each session, the FC network for each participant was constructed using regularized partial correlations estimated with graphical LASSO with a common regularization parameter $\lambda$ of 0.1 [59]. This resulted in sparse, weighted, undirected networks, with each edge representing the unique relation between ROIs conditional on all other ROIs in the network. Both positive and negative partial correlations were retained. Partial correlations were used instead of bivariate correlations because partial correlations can be easily regularized to form a sparse FC network, which is needed for NCT analyses. Additionally, partial correlations estimate the unique relationships between two variables controlling for all other variables in the dataset, which better correspond to the coefficients of a dynamic linear system than bivariate correlations (which capture the marginal linear relationship between two variables).

### Network Control Theory (NCT)

**Fig. 1:**
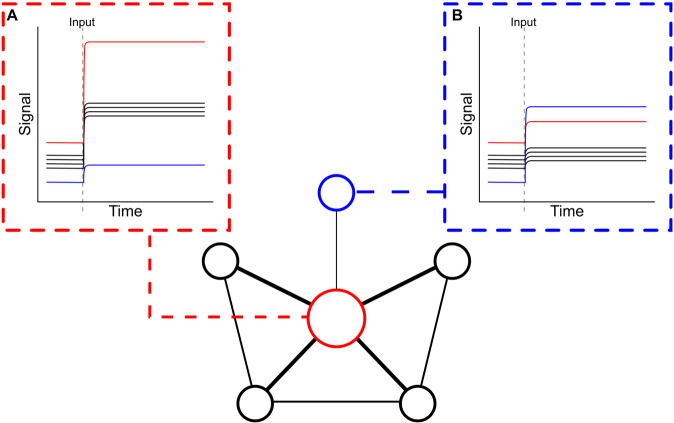
A conceptual illustration of network control theory (NCT). The central network represents a dynamical system of six nodes, with edge thickness representing the strength of each connection. **A** Shows the effect on the output signal of the entire system when an input is made to the red node. This input leads to the largest increase in the signal of the red node, a moderately large increase in the signal of the 4 black nodes, and a small increase in the signal of the blue node. This is indicative of the red node having high average controllability, as overall the system responds strongly to input to the red node. **B** Shows the effect on the entire system when an input is made to the blue node. This input leads to a large increase in the signal of the blue node and only small increases in the signal of the red and black nodes. This is indicative of the blue node having high modal controllability, as the blue node itself is difficult to reach via other nodes. The blue node also has low average controllability, as input to the blue node does not impact the rest of the system strongly.

NCT describes brain activity as the following linear dynamical system:

$$x\left( {t + 1} \right) = Ax\left( t \right) + Bu(t)$$

where $t$ is time, $x$ is the multidimensional signal from functional MR imaging, $A$ is a connectome inferred from either structural or functional imaging data, $u$ represents arbitrary inputs, and $B$ describes how those inputs impact the functional signal. Traditional graph theoretic approaches to network neuroscience have focused on the topology of $A$ (the connectome). NCT expands on these approaches by examining how inputs to specific regions or networks, as a result of endogenous fluctuations in BOLD signal or brain response to external task stimuli, interact with the underlying connectome to impact the functional signal (i.e., BOLD response) in the rest of the brain. Here, we quantified two common NCT metrics: average controllability and modal controllability. Average controllability is a measure of how much an input to a given ROI (or set of ROIs) results in a change in the output signal of the overall system [21, 22]. Regions with high average controllability have a larger impact on activity in the rest of the brain as compared to those with low average controllability. Theoretically, these regions are capable of steering activity into and out of states using less energy than those with lower average controllability, thus they are primary drivers of changes in activity patterns across the entire brain. Here, we quantified how input to a network of ROIs (e.g., the default mode network or the fronto-parietal network) impacted average BOLD signal across the brain. Average controllability for each network was calculated as the mean average controllability of all ROIs within that network. Modal controllability is a measure of the ability of an input to a given ROI (or set of ROIs) to drive the whole brain into states that are difficult to reach [21, 22]. Theoretically, difficult-to-reach brain states are thought to be states entered under circumstances of high cognitive demands [60, 61]. This suggests that higher modal controllability corresponds to using less energy to enter these difficult-to-reach brain states when performing cognitively demanding tasks. Similar to average controllability, modal controllability for each network was calculated by taking the mean modal controllability of all ROIs within that network. The controllability metrics were calculated using the netcontrol R package [62]. Figure 1 provides a conceptual schematic for the NCT approach.

NCT is most commonly applied to the study of structural connectomes under the assumption that the structural connectome underlies the dynamical system of brain function, with stronger structural connections corresponding to greater functional relations [21]. While applying NCT to structural connectomes has been a powerful tool for analyzing functional dynamics, it precludes the ability to probe how the underlying connectome impacts BOLD response in situations in which the functional connectome itself dynamically changes (e.g., across cognitive contexts or after treatment). Therefore, we apply NCT to the functional connectome here, allowing us to assess *functional controllability*, in contrast to the more static structural controllability assessed with previous uses of NCT.

### Statistical analysis

A set of mixed effects models (one for each scan context for each network) was used to examine the differences in network-level average and modal controllability between TD children (using both scan sessions coded as placebo) and children with ADHD (both on MPH and on placebo). A second set of mixed effects models (one for each scan context for each network) was used to examine the effects of MPH on average and modal controllability in children with ADHD (on-off MPH). The two model sets were estimated separately since the study design was not fully crossed, as TD children were not administered MPH. Both model types controlled for age, biological sex, and in-scanner motion (average framewise displacement without notch filtering, before censoring and processing). They additionally used a random intercept at the level of the participant to account for participant-specific differences (which in turn was aggregated over multiple TD sessions, when available). This resulted in a total of 156 models (2 model sets x 2 metrics x 3 scan contexts x 13 networks). All models were fit using the lme4 R package [63]. We chose to run all possible models instead of focusing on specific networks evidenced to be related to ADHD and impacted by MPH due to the novelty of applying NCT to functional data. Thus, due to the exploratory nature of this preliminary study, we did not apply a multiple comparisons correction.

## Results

### Average controllability

**Fig. 2:**
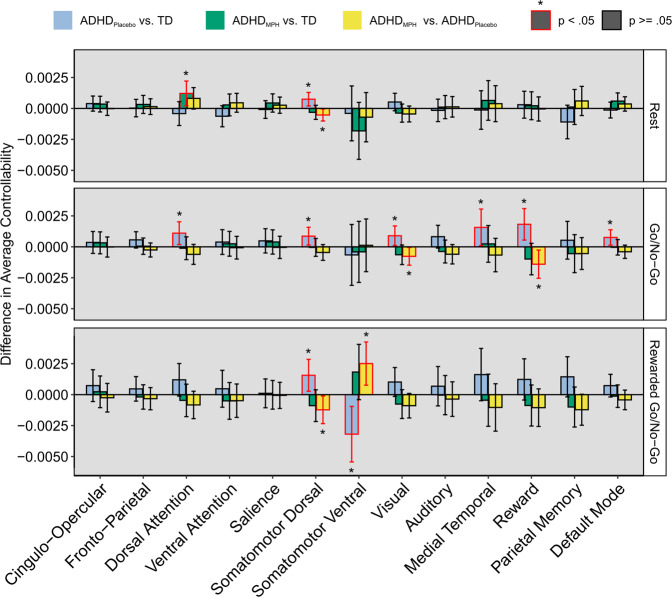
Difference in average controllability between ADHD on placebo and TD (blue bars), ADHD on MPH and TD (green bars), and within ADHD (yellow bars) for the three functional scan contexts (rest, go/no-go, rewarded go/no-go). Children with ADHD on placebo exhibited increased average controllability in the somatomotor dorsal network during rest, the dorsal attention, somatomotor dorsal, visual, medial temporal, reward, and default mode networks during the go/no-go task, and in the somatomotor dorsal network during the rewarded go/no-go task. Children with ADHD on placebo exhibited decreased average controllability in the somatomotor ventral network during the rewarded go/no-go task. During rest, children with ADHD on MPH exhibited higher average controllability in the dorsal attention network. For all cases in which children with ADHD on placebo exhibited significantly different average controllability compared to TD children, those differences were no longer significant when comparing children with ADHD on MPH and TD children. This was supported by significant within-ADHD effects of MPH for the somatomotor dorsal network during rest, visual and reward networks during the go/no-go task, and the somatomotor dorsal and somatomotor ventral networks during the rewarded go no-go task. Bars correspond to the regression estimate of the relevant difference, controlling for age, biological sex, and in-scanner motion. Error bars correspond to 95% confidence intervals. Red outline and asterisk indicate statistical significance at *p* < 0.05.

First, we quantified average controllability of individual functional networks, or how reactive the whole brain was to inputs to each network. During rest, children with ADHD on placebo exhibited increased average controllability in the somatomotor dorsal network relative to TD children ($\beta = 0.0007,p = 0.01$). This group difference was not significant when children with ADHD were on MPH (TD vs. ADHD on MPH: $\beta = - 0.0003,p = 0.29$), and there was a corresponding significant within-participant reduction in average controllability associated with MPH ($\beta = - 0.0005,p = 0.04$). Additionally, children with ADHD on MPH had significantly increased average controllability relative to TD children in the dorsal attention network $(\beta = 0.0012,p = 0.02)$; this difference was not present in children with ADHD on placebo. All other effects were non-significant (all *p-*values > 0.07; Fig. 2).

During the go/no-go task, children with ADHD on placebo exhibited increased average controllability in the dorsal attention ($\beta = 0.001,p = 0.02$), somatomotor dorsal ($\beta = 0.001,p = 0.02$), visual ($\beta = 0.0008,p = 0.03$), medial temporal (*β* = 0.002, *p* = 0.04), reward ($\beta = 0.002,p = 0.007$), and default mode (*β* = 0.0008, *p* = 0.02) networks relative to TD children. These group differences were not present when children with ADHD were on MPH (all *p-*values > 0.11). There were corresponding significant within-participant reductions in average controllability associated with MPH for the visual ($\beta = - 0.0008,p = 0.04$) and reward ($\beta = 0.0014,p = 0.02$) networks. All other effects were non-significant (all *p-*values > 0.05; Fig. 2).

During the rewarded go/no-go task, children with ADHD on placebo exhibited increased average controllability relative to TD children in the somatomotor dorsal network ($\beta = 0.0016,p = 0.02$) and decreased average controllability in the somatomotor ventral network ($\beta = - 0.003,p = 0.01$), with no significant differences between TD children and children with ADHD on MPH in either of these networks (both *p-*values > 0.10). In both cases there was a significant within-participant effect of average controllability associated with MPH ($\beta = - 0.0012,p = 0.04$ and $\beta = 0.002,p = 0.007$ respectively). All other effects were non-significant (all *p-*values > 0.06; Fig. 2).

For parameter estimates, see Tables S6, S7, and S8 in Supplemental Information.

### Modal controllability

**Fig. 3:**
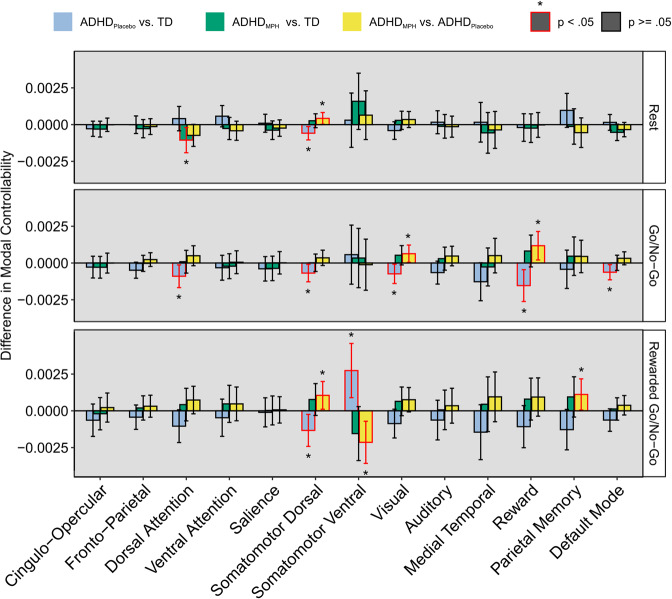
Difference in modal controllability between ADHD on placebo and TD (blue bars), ADHD on MPH and TD (green bars), and within ADHD (yellow bars) for the three functional scan contexts (rest, go/no-go, rewarded go/no-go). Children with ADHD on placebo exhibited decreased modal controllability in the somatomotor dorsal network during rest, the dorsal attention, somatomotor dorsal, visual, reward, and default mode networks during the go/no-go task, and in the somatomotor dorsal network during the rewarded go/no-go task. Children with ADHD on placebo exhibited increased average controllability in the somatomotor ventral network during the rewarded go/no-go task. During rest, children with ADHD on MPH exhibited lower modal controllability in the dorsal attention network. For all cases in which children with ADHD on placebo exhibited significantly different modal controllability compared to TD children, those differences were no longer significant when comparing children with ADHD on MPH and TD children. This was supported by significant within-ADHD effects of MPH for the somatomotor dorsal network during rest, visual and reward networks during the go/no-go task, and the somatomotor dorsal and somatomotor ventral networks during the rewarded go no-go task. Bars correspond to the regression estimate of the relevant difference, controlling for age, biological sex, and in-scanner motion. Error bars correspond to 95% confidence intervals. Red outline and asterisk indicate statistical significance at *p* < 0.05.

Next, we quantified modal controllability of individual functional networks, or how easy it was for inputs to each network to drive the whole brain toward difficult-to-reach states. During rest, children with ADHD on placebo exhibited decreased modal controllability in the somatomotor dorsal network ($\beta = - 0.0006,p = 0.01$). This group difference was not significant when children with ADHD were on MPH (TD vs. ADHD on MPH: $\beta = 0.0003,p = 0.28$). There was a corresponding significant increase in modal controllability of the somatomotor dorsal network in children with ADHD on MPH compared to on placebo ($\beta = 0.0004,p = 0.04$). Additionally, children with ADHD on MPH had significantly decreased modal controllability relative to TD children in the dorsal attention network $(\beta = - 0.001,p = 0.02)$; this difference was not present in children with ADHD on placebo. All other effects were non-significant (all *p-*values > 0.06; Fig. 3).

During the go/no-go task, children with ADHD on placebo exhibited decreased modal controllability in the dorsal attention ($\beta = - 0.0009,p = 0.03$), somatomotor dorsal ($\beta = - 0.0007,p = 0.03$), visual ($\beta = - 0.0007,p = 0.03$), reward ($\beta = - 0.0015,p = 0.006$), and default mode ($\beta = - .0006,p = 0.02$) networks relative to TD children. These group differences were not present when children with ADHD were on MPH (all *p-*values > 0.12). Additionally, there was a corresponding significant increase in modal controllability in children with ADHD on MPH compared to on placebo for the visual ($\beta = 0.0006,p = 0.04$) and reward ($\beta = 0.0012,p = 0.02$) networks. All other effects were non-significant (all *p-*values > 0.05; Fig. 3).

During the rewarded go/no-go task, children with ADHD on placebo exhibited decreased modal controllability in the somatomotor dorsal network ($\beta = - 0.0013,p = 0.02$) and increased modal controllability in the somatomotor ventral network ($\beta = 0.003,p = 0.005$) relative to TD children. These group differences were not present when children with ADHD were on MPH (both *p-*values > 0.10). There was a corresponding significant within ADHD effect of MPH for the somatomotor dorsal and somatomotor ventral networks (*β* = 0.001, *p* = 0.04; *β* = −0.002, *p* = 0.005 respectively). Additionally, there was a significant increase in modal controllability in children with ADHD on MPH compared to on placebo of the parietal memory network $(\beta = 0.001,p = 0.046)$, though there were no significant differences between TD children and children with ADHD on MPH or on placebo in the parietal memory network (both *p-*values > 0.05). All other effects were non-significant (all *p-*values > 0.07; Fig. 3).

For parameter estimates, see Tables S9, S10, and S11 in Supplemental Information.

## Discussion

In this study, we investigated the functional controllability of brain networks across different cognitive contexts in medication-naïve children with ADHD, as well as how controllability changed after MPH administration. Compared to TD children, children with ADHD who were not on MPH exhibited higher average controllability and lower modal controllability in several networks, including the default mode, dorsal attention, reward, somatomotor, and visual networks. These differences were reduced following MPH administration.

The networks with altered controllability in children with ADHD on placebo are those that prior research has identified as having disrupted FC in ADHD [25, 26]. The overall pattern of higher average controllability and lower modal controllability indicates that the brains of children with ADHD were more reactive to inputs yet less capable of entering difficult-to-reach brain states that have been associated with highly demanding cognitive processes [60, 61]. Higher average controllability, or increased neural reactivity, distributed across several networks could underlie greater susceptibility to distractions. This is consistent with theories positing that attention lapses in ADHD are caused by disruptions in task-relevant network functioning due to default mode network interference [64]. The current findings indicate that the mechanism through which the default mode network may interfere with task-relevant network functioning may be through greater global reactivity of both default mode and task-relevant networks to distracting stimuli. Our finding of reduced modal controllability in the same networks indicates that in addition to greater reactivity, children with ADHD may have more difficulty entering into states characterized by greater ability to sustain attention or to complete cognitively demanding tasks. This is consistent with recent literature observing that children with ADHD are less likely to enter or sustain brain states associated with successful cognitive control [18].

Controllability between children with ADHD and TD children differed based on cognitive context, with a greater number of functional networks showing significant differences across groups during the tasks relative to rest, in particular during the go/no-go task. In other words, in the more constrained (and cognitively demanding) task context, group differences in controllability were more readily observable. Prior literature has observed larger group differences [31, 34, 35] and stronger relationships between FC and behavior [32, 33] during more difficult compared to easier conditions of cognitive tasks. While the pattern of results was similar during the two go/no-go tasks, there were fewer significant group differences during the rewarded as compared to the unrewarded go/no-go task. This could be because the variance in controllability was higher during the rewarded go/no-go task. This increased variability may be due to individual differences in the motivating nature of the rewards [65], however there was little difference in the variability of task performance between the regular and rewarded go/no-go tasks (SD for go/no-go d’ - TD: 0.82, ADHD Placebo: 0.92, ADHD MPH: 0.99; SD for rewarded go/no-go d’- TD: 0.85, ADHD Placebo: 1.02, ADHD MPH: 0.88).

Consistent with our hypotheses, MPH administration generally eliminated differences in controllability between ADHD and TD groups, with the only significant difference in the controllability metrics of children with ADHD on MPH compared to TD children localized to the dorsal attention network during rest. This is consistent with prior literature that MPH reduces differences between children with ADHD and TD children for brain activity and FC of specific connections during cognitive tasks [13, 66, 67]. Notably, changes in FC due to MPH during rest are not consistent across studies [16]. This is congruous with our finding that there were fewer differences in controllability across groups or changes after MPH administration during the resting state and indicates that probing FC and network topology during cognitive tasks may reveal more consistent effects of MPH.

There are several limitations to the present study. First, the small sample size, particularly of the children with ADHD (18 or 19 depending on scan context), limits our power to detect group differences, as does the reduction in number of timepoints due to the censoring of high motion volumes. Related to this, differences in findings between scan contexts could in part be due to differences in the number of timepoints going into each task (maxima are 150 for rest, 195 for go/no-go, and 185 for rewarded go/no-go before censoring). Second, the study was designed to evaluate acute response to MPH administration, rather than longer term behavioral response to MPH treatment, which curtailed our ability to identify MPH responders using typically applied criteria (i.e., reduction in symptoms). This limitation, combined with our small sample size, led us to define acute behavioral response on a task-by-task basis to balance sample size with the behavioral response criteria, which in turn led to some participants being considered responders for one task and non-responders for other tasks. We tested a stricter behavioral response criterion, defining responders as those with reductions in head motion across all three tasks, which reduced the sample size to 13. These models *(*Supplemental Information, Figures S1 and S2) show similar patterns of effects, albeit with lower power to detect significant differences. Third, previous work has shown that excluding participants based on high motion can bias the clinical characteristics of high motion populations [68]. We conducted analyses to assess any differences in ADHD symptom severity and/or performance on our tasks between our included and excluded participants (see Supplemental Information, Tables S4 and S5) and found no significant differences between included/excluded participants. However, given the small sample size, and particularly the small number of excluded participants (*n* = 12), the analyses we performed are likely underpowered to detect group differences, and the pattern of effect sizes suggest that the included participants had slightly lower hyperactivity/impulsivity severity and slightly better performance on both go/no-go tasks. Induced bias due to exclusion criteria is particularly important to consider in high motion populations such as the one under study, and it is likely that our included participants have less severe symptoms than the entire population of children with ADHD. It should be noted, however, that having less severe symptoms is likely to attenuate any effects, rather than lead to upwardly biased estimates. Given these limitations, although the observed networks in which we observed group differences are consistent with prior literature, our results should be taken as exploratory, rather than confirmatory. Future research is needed to confirm the specific networks that show differences in functional controllability as related to ADHD, especially in larger samples and with prolonged treatment to better assess MPH response.

In summary, our findings suggest that the functional dynamics of children with ADHD are characterized by instability in a context of high cognitive demands. Children with ADHD were less likely to enter difficult-to-reach states that may underlie successful response inhibition (lower modal controllability), while they were more likely to be “pushed out” of those states by non-task-related stimuli (higher average controllability). Furthermore, we identified a possible network-level mechanism through which MPH, a dopamine and noradrenaline transporter blocker, may reduce symptom severity in ADHD: by increasing stability and the ability to enter states necessary for cognitive control. This is consistent with recent work reporting that increased dopamine D1 receptor gene expression was related to increased stability of brain states related to cognitive control, in that case working memory representations [69]. Finally, our study demonstrates the utility of using NCT with functional connectomes to characterize the functional consequences of disrupted connectivity.

## Supplementary information

Supplemental Materials

## Data Availability

Data and code used in this manuscript is available upon request to the senior author (JRC).
