# Supplementary material for: Effect of methylphenidate on functional controllability: a preliminary study in medication-naïve children with ADHD
Source: Transl Psychiatry. 2022 Dec 17;12:518. doi: 10.1038/s41398-022-02283-4 (PMC9759578; doi:10.1038/s41398-022-02283-4)
Supplement: Supplementary file 1 — Supplemental Materials [file 41398_2022_2283_MOESM1_ESM.docx]

**Supplemental Information for “Effect of methylphenidate on functional controllability: a preliminary study in medication-
naïve children with ADHD”**

**Supplemental Methods**

**Subject Inclusion and Exclusion Criteria**

To be eligible, participants were required to be between the ages of 8 years, 0 months and 12 years, 11 months, 30 days. Children with attention-deficit/hyperactivity disorder (ADHD) were required to meet diagnostic criteria for ADHD according to the Diagnostic Interview Schedule for Children Version IV (DISC-IV) ^1^ and the Conners 3^rd^ Edition Parent and Teacher Rating Scales ^2^. Given evidence the DISC-IV assesses behavior across multiple settings (i.e., home and school), children were diagnosed with ADHD if they met the following criteria: 1) full diagnostic criteria for ADHD on the DISC-IV or 2) intermediate diagnostic criteria for ADHD on the DISC-IV and full diagnostic criteria for ADHD on the Conners 3^rd^ Edition Parent and/or Teacher Rating Scales. In addition to using the DISC-IV and the Conners Rating Scales to establish categorical diagnosis of ADHD, the Swanson, Nolan, and Pelham Rating Scale (SNAP-IV) ^3^ was administered to assess dimensional ADHD symptoms. Children with ADHD were also required to be medication-naïve and to complete a physical examination to ensure that they could safely be administered methylphenidate (MPH). Typically developing (TD) children were required to have no history or presence of developmental or psychiatric disorders, and no history or presence of ADHD in immediate family members (i.e., parents or siblings). Exclusion criteria for all children was assessed via the DISC-IV, as well as a review of medical and developmental history. Additional criteria for exclusion was as follows: (1) diagnosis of intellectual disability, developmental speech/language disorder, reading disability, autism spectrum disorder, or a pervasive developmental disorder; (2) visual impairment that could not be corrected-to-normal, including color blindness; (3) neurologic disorder (e.g., epilepsy, cerebral palsy, traumatic brain injury, Tourette syndrome); (4) documented hearing impairment characterized by **>**25 decibel loss in either ear; (5) had already gone through puberty; for girls this means that in addition to other developmental stages they had experienced menarche; and (6) medical contraindication to MRI (e.g., implanted electrical devices, dental braces). In addition, children were excluded if their Full Scale IQ score as determined using the Weschler Intelligence Scale for Children, Fifth Edition (WISC-V) ^4^ was < 85 or if their Word Reading subtest score from the Wechsler Individual Achievement Test, 3^rd^ Edition (WIAT-III) ^5^ was < 85 on the Word Reading subtest, regardless of Full Scale IQ score.

**Minimal Preprocessing with FMRIPREP**

Results included in this manuscript come from preprocessing performed using fMRIPrep 1.5.0 ^6,7^, which is based on Nipype 1.2.2 ^8,9^. Text is taken directly from fMRIPrep output reports with minimal modification for clarity and readability.

**Anatomical data preprocessing**

All T1-weighted (T1w) images were corrected for intensity non-uniformity (INU) with N4BiasFieldCorrection ^10^, distributed with ANTs 2.2.0 ^11^.The T1w-reference was then skull-stripped with a Nipype implementation of the antsBrainExtraction.sh workflow (from ANTs), using OASIS30ANTs as target template. Brain tissue segmentation of cerebrospinal fluid (CSF), white-matter (WM) and gray-matter (GM) was performed on the brain-extracted T1w using fast ^12^. A T1w-reference map was computed after registration of two T1w images (after INU-correction) using mri_robust_template ^13^. Brain surfaces were reconstructed using recon-all ^14^, and the brain mask estimated previously was refined with a custom variation of the method to reconcile ANTs-derived and FreeSurfer-derived segmentations of the cortical gray-matter of Mindboggle ^15^ . Volume-based spatial normalization to one standard space was performed through nonlinear registration with antsRegistration (ANTs 2.2.0), using brain-extracted versions of both T1w-reference and the T1w template. The following template was selected for spatial normalization: ICBM 152 Nonlinear Asymmetrical template version 2009c (MNI152NLin2009cAsym) ^14^.

**Functional data preprocessing**

For each of the BOLD runs (across all participants, sessions, and tasks), the following preprocessing was performed. First, a reference volume and its skull-stripped version were generated using a custom methodology of fMRIPrep. A deformation field to correct for susceptibility distortions was estimated based on fMRIPrep’s fieldmap-less approach. The deformation field is that resulting from co-registering the BOLD reference to the same-subject T1w-reference with its intensity inverted ^17,18^. Registration was performed with antsRegistration (ANTs 2.2.0), and the process was regularized by constraining deformation to be nonzero only along the phase-encoding direction, and modulated with an average fieldmap template ^19^. Based on the estimated susceptibility distortion, an unwarped BOLD reference was calculated for a more accurate co-registration with the anatomical reference. The BOLD reference was then co-registered to the T1w-reference using bbregister (FreeSurfer), which implements boundary-based registration ^20^. Co-registration was configured with six degrees of freedom. Head-motion parameters with respect to the BOLD reference (transformation matrices, and six corresponding rotation and translation parameters) were estimated before any spatiotemporal filtering using mcflirt ^21^. BOLD runs were slice-time corrected using 3dTshift from AFNI 20160207 ^22^. The BOLD time-series were resampled to surfaces on the fsaverage5 space. The BOLD time-series (including slice-timing correction) were resampled onto their original, native space by applying a single, composite transform to correct for head-motion and susceptibility distortions. The BOLD time-series were then resampled into standard space, generating a preprocessed BOLD run in MNI152NLin2009cAsym space. First, a reference volume and its skull-stripped version were generated using a custom methodology of fMRIPrep. The following confounding time-series were calculated based on the preprocessed BOLD and used here: framewise displacement (FD) and three region-wise global signals (CSF, WM, and the whole-brain GM mask). FD was calculated for each functional run using its implementation in Nipype (following the definitions by ^23^). The head-motion estimates calculated in the correction step were also placed within the corresponding confounds file. The confound time-series derived from head-motion estimates and global signals were expanded with the inclusion of temporal derivatives and quadratic terms for each ^24^. All resamplings were performed with a single interpolation step by composing all the pertinent transformations (i.e., head-motion transform matrices, susceptibility distortion correction, and co-registrations to anatomical and output spaces). Gridded (volumetric) resamplings were performed using antsApplyTransforms (ANTs), configured with Lanczos interpolation to minimize the smoothing effects of other kernels ^25^. Non-gridded (surface) resamplings were performed using mri_vol2surf (FreeSurfer).

Many internal operations of fMRIPrep use Nilearn 0.5.2 ^26^, mostly within the functional processing workflow. For more details of the pipeline, see the section corresponding to workflows in fMRIPrep’s documentation.

**Missing ROIs**

For analyses, a functional atlas consisting of 300 spherical ROIs with 4 mm radii was used ^27^. Due to limited field of view, data from 16 ROIs was not collected in all participants, thus these ROIs were not included in analyses. See Table S1 for coordinates and assigned networks of the missing ROIs.

**Table S1:** Network Labels and MNI Coordinates for Missing ROIs. The 16 ROIs in this table were removed from the analysis due to not having complete data across all subjects.

| ROI Number | Network Labels | MNI x | MNI y | MNI z |
| --- | --- | --- | --- | --- |
| 6 | Unassigned | 51.79 | -34.17 | -27.23 |
| 10 | Unassigned | -50.06 | -7.09 | -39.24 |
| 77 | Default Mode | -68.3 | -41.41 | -5.14 |
| 85 | Default Mode | -43.58 | 11.99 | -34.15 |
| 234 | Medial Temporal | -31.13 | -9.99 | -36.32 |
| 274 | Cerebellum- Unassigned | 32 | -49 | -51 |
| 275 | Cerebellum- Unassigned | -13 | -52 | -50 |
| 276 | Cerebellum- Unassigned | 14 | -48 | -52 |
| 277 | Cerebellum- Default Mode | -32 | -78 | -38 |
| 281 | Cerebellum- Default Mode | -5.72 | -50.8 | -40.84 |
| 282 | Cerebellum- Default Mode | 8 | -50 | -40 |
| 286 | Cerebellum- Fronto-Parietal | -34 | -72.01 | -48 |
| 287 | Cerebellum- Fronto-Parietal | 34 | -72 | -48 |
| 293 | Cerebellum- Cingulo-Opercular | -34 | -42 | -44 |
| 295 | Cerebellum- Somatomotor Dorsal | -6 | -74 | -42 |
| 296 | Cerebellum- Somatomotor Dorsal | 7.5 | -72 | -39 |

**Analysis of Relationship Between Head Motion and ADHD Symptom Severity**

We assessed the relationship between head motion and ADHD symptom severity to determine whether we were more likely to exclude participants with a particular subtype of ADHD based on our inclusion criteria. To do so, we fit a series of linear regression models separately for each scan context. Table S2 contains the estimates from models predicting average raw framewise displacement (FD; before notch filtering, data processing, or censoring) from inattention and hyperactivity/impulsivity severity for the children with ADHD in the placebo session only. Table S3 contains the estimates from models predicting the change in average FD from placebo to MPH sessions from inattention and hyperactivity/impulsivity severity. For both analyses, we included all participants with ADHD, regardless of whether they were excluded from the main analyses due to excessive head motion during the MRI scans. All p-values are uncorrected for multiple comparisons. For both the analysis of raw head motion during the placebo session and the analysis of change in head motion due to MPH, there were no significant effects of ADHD symptom severity.

**Table S2:** Linear Regression Models Predicting Raw Framewise Displacement (FD) separately by Task During Placebo Session using ADHD Symptom Severity

|  | **Estimate** | **Std. Error** | **t-value** | **p-value** |
| --- | --- | --- | --- | --- |
| **Resting State** |  |  |  |  |
| Intercept | -0.12 | 0.22 | 0.56 | 0.59 |
| Inattention Severity | -0.24 | 0.23 | -1.09 | 0.29 |
| Hyp/Imp Severity | 0.21 | 0.23 | 0.91 | 0.38 |
| **Go/No-Go** |  |  |  |  |
| Intercept | 0.02 | 0.22 | 0.11 | 0.92 |
| Inattention Severity | -0.15 | 0.24 | -0.62 | 0.54 |
| Hyp/Imp Severity | -0.09 | 0.24 | -0.40 | 0.69 |
| **Rewarded Go/No-Go** |  |  |  |  |
| Intercept | -0.07 | 0.18 | -0.37 | 0.72 |
| Inattention Severity | -0.22 | 0.20 | -1.10 | 0.28 |
| Hyp/Imp Severity | 0.003 | 0.20 | 0.18 | 0.99 |

Estimates are in standardized $(\beta)$ metric (i.e., change in SD units for 1 SD change in predictor).

**Table S3:** Linear Regression Models Predicting Change in Framewise Displacement (FD) from Placebo to MPH Sessions using ADHD Symptom Severity

|  | **Estimate** | **Std. Error** | **t-value** | **p-value** |
| --- | --- | --- | --- | --- |
| **Resting State** |  |  |  |  |
| Intercept | -0.11 | 0.23 | -0.48 | 0.64 |
| Inattention Severity | -0.21 | 0.23 | -0.87 | 0.40 |
| Hyp/Imp Severity | -0.10 | 0.23 | -0.44 | 0.67 |
| **Go/No-Go** |  |  |  |  |
| Intercept | -0.19 | 0.20 | -0.95 | 0.35 |
| Inattention Severity | 0.15 | 0.20 | 0.77 | 0.45 |
| Hyp/Imp Severity | 0.10 | 0.19 | 0.50 | 0.62 |
| **Rewarded Go/No-Go** |  |  |  |  |
| Intercept | 0.01 | 0.14 | 0.08 | 0.94 |
| Inattention Severity | 0.05 | 0.14 | 0.33 | 0.75 |
| Hyp/Imp Severity | 0.05 | 0.14 | 0.39 | 0.70 |

Estimates are in standardized $(\beta)$ metric (i.e., change in SD units for 1 SD change in predictor).

**Analysis of Differences in ADHD Symptom Severity and Task Performance due to Motion Exclusion Criteria**

Previous work has shown that excluding participants on the basis of in-scanner motion can bias clinical and behavioral measures in the analyzed sample, particularly when the underlying clinical phenomena is related to motion ^28^. To assess this potential bias we estimated a series of linear regression models predicting symptom severity and go/no-go performance in children with ADHD by exclusion status (coded 1 [excluded; n = 12]/0 [included; n = 23]). Table S4 contains the regression estimates for exclusion status predicting inattention severity and hyperactivity severity, while Table S5 contains the regression estimates for exclusion status predicting d’ (averaged across runs) in the regular and rewarded go/no-go tasks separately. For both sets of analyses, excluded status was not significantly related to any of the tested outcomes, although numerically participants with ADHD who were included had less severe hyperactivity/impulsivity symptoms (mean included = 1.38, mean excluded =1.59) and performed better on the regular go/no-go task (mean included = 1.81, mean excluded = 1.76).

**Table S4:** Linear Regression Models Predicting ADHD Symptom Severity from Exclusion Status

|  | **Estimate** | **Std. Error** | **t-value** | **p-value** |
| --- | --- | --- | --- | --- |
| **Inattention Severity** |  |  |  |  |
| Intercept | 0.03 | 0.22 | 0.12 | 0.91 |
| Excluded Status | -0.07 | 0.37 | -0.2 | 0.85 |
| **Hyp/Imp Severity** |  |  |  |  |
| Intercept | -0.11 | 0.22 | -0.51 | 0.61 |
| Excluded Status | 0.29 | 0.35 | 0.82 | 0.41 |

Estimates are in standardized $(\beta)$ metric (i.e., change in SD units).

**Table S5:** Linear Regression Models Predicting Go/No-Go d’ from Exclusion Status

|  | **Estimate** | **Std. Error** | **t-value** | **p-value** |
| --- | --- | --- | --- | --- |
| **Go/No-Go d’** |  |  |  |  |
| Intercept | 0.09 | 0.21 | 0.41 | 0.70 |
| Excluded Status | -0.25 | 0.36 | -0.70 | 0.49 |
| **Rewarded Go/No-Go d’** |  |  |  |  |
| Intercept | 0.02 | 0.22 | 0.09 | 0.93 |
| Excluded Status | -0.06 | 0.38 | -0.16 | 0.87 |

Estimates are in standardized $(\beta)$ metric (i.e., change in SD units).

**Supplemental Results**

The following tables contain parameter estimates, t-values, and significance values for the multi-level models reported in the *Main Text*. Tables S6, S7, and S8 contain results from the models predicting average controllability (Figure 2 in the *Main Text*), while Tables S9, S10, and S11 contain results from the models predicting modal controllability (Figure 3 in the *Main Text*).

**Results Tables**

| **Table S6:** Average Controllability Effect Estimates for the Resting State. | | | | | | | | | |
| --- | --- | --- | --- | --- | --- | --- | --- | --- | --- |
|  | ADHD_Placebo_ vs. TD | | | ADHD_MPH_ vs. TD | | | ADHD_Placebo_ vs. ADHD_MPH_ | | |
| **Network** | **Estimate** | **t value** | **Pr(>\|t\|)** | **Estimate** | **t value** | **Pr(>\|t\|)** | **Estimate** | **t value** | **Pr(>\|t\|)** |
| Auditory | -0.0002 | -0.33 | 0.74 | 0.0001 | 0.23 | 0.82 | 0.0001 | 0.31 | 0.76 |
| Cingulo-Opercular | 0.0004 | 1.26 | 0.21 | 0.0004 | 1.08 | 0.28 | 0 | -0.07 | 0.94 |
| Default Mode | -0.0001 | -0.41 | 0.69 | 0.0006 | 1.77 | 0.08 | 0.0004 | 1.23 | 0.23 |
| Dorsal Attention | -0.0004 | -0.85 | 0.40 | **0.0012** | **2.39** | **0.02** | 0.0008 | 1.84 | 0.07 |
| Fronto-Parietal | 0 | 0.08 | 0.94 | 0.0003 | 0.87 | 0.39 | 0.0001 | 0.46 | 0.65 |
| Medial Temporal | -0.0001 | -0.16 | 0.88 | 0.0006 | 0.79 | 0.43 | 0.0004 | 0.52 | 0.60 |
| Parietal Memory | -0.0011 | -1.58 | 0.12 | 0.0001 | 0.16 | 0.87 | 0.0006 | 1.01 | 0.32 |
| Reward | 0.0003 | 0.54 | 0.59 | 0.0002 | 0.38 | 0.71 | 0 | -0.09 | 0.93 |
| Salience | -0.0001 | -0.23 | 0.82 | 0.0004 | 1.17 | 0.25 | 0.0003 | 0.79 | 0.43 |
| Somatomotor Dorsal | **0.0007** | **2.68** | **0.009** | -0.0003 | -1.08 | 0.29 | **-0.0005** | **-2.15** | **0.04** |
| Somatomotor Ventral | -0.0004 | -0.35 | 0.73 | -0.0018 | -1.54 | 0.13 | -0.0007 | -0.70 | 0.49 |
| Ventral Attention | -0.0006 | -1.45 | 0.15 | 0.0003 | 0.63 | 0.53 | 0.0005 | 1.17 | 0.25 |
| Visual | 0.0005 | 1.44 | 0.16 | -0.0004 | -1.01 | 0.32 | -0.0004 | -1.37 | 0.18 |
| Bold indicates significant effects with p-value < .05, uncorrected. | | | | | | | | | |

| **Table S8:** Average Controllability Effect Estimates for the Rewarded Go/No-Go Task. | | | | | | | | | |
| --- | --- | --- | --- | --- | --- | --- | --- | --- | --- |
|  | ADHD_Placebo_ vs. TD | | | ADHD_MPH_ vs. TD | | | ADHD_Placebo_ vs. ADHD_MPH_ | | |
| **Network** | **Estimate** | **t value** | **Pr(>\|t\|)** | **Estimate** | **t value** | **Pr(>\|t\|)** | **Estimate** | **t value** | **Pr(>\|t\|)** |
| Auditory | 0.0007 | 0.84 | 0.40 | 0 | -0.05 | 0.96 | -0.0004 | -0.50 | 0.62 |
| Cingulo-Opercular | 0.0007 | 1.11 | 0.27 | 0.0002 | 0.35 | 0.73 | -0.0003 | -0.42 | 0.67 |
| Default Mode | 0.0007 | 1.56 | 0.13 | -0.0001 | -0.27 | 0.79 | -0.0004 | -1.05 | 0.30 |
| Dorsal Attention | 0.0012 | 1.79 | 0.08 | -0.0005 | -0.70 | 0.49 | -0.0008 | -1.47 | 0.15 |
| Fronto-Parietal | 0.0005 | 0.92 | 0.36 | -0.0002 | -0.38 | 0.71 | -0.0003 | -0.72 | 0.48 |
| Medial Temporal | 0.0016 | 1.50 | 0.14 | -0.0005 | -0.42 | 0.68 | -0.001 | -1.06 | 0.30 |
| Parietal Memory | 0.0014 | 1.74 | 0.09 | -0.001 | -1.21 | 0.23 | -0.0012 | -1.91 | 0.06 |
| Reward | 0.0012 | 1.45 | 0.15 | -0.0009 | -1.04 | 0.31 | -0.0011 | -1.36 | 0.18 |
| Salience | 0.0001 | 0.18 | 0.86 | 0 | -0.02 | 0.98 | -0.0001 | -0.11 | 0.91 |
| Somatomotor Dorsal | **0.0016** | **2.38** | **0.02** | -0.0009 | -1.36 | 0.18 | **-0.0012** | **-2.15** | **0.04** |
| Somatomotor Ventral | **-0.0032** | **-2.79** | **0.007** | 0.0018 | 1.60 | 0.11 | **0.0025** | **2.82** | **0.007** |
| Ventral Attention | 0.0005 | 0.62 | 0.54 | -0.0005 | -0.67 | 0.51 | -0.0005 | -0.71 | 0.48 |
| Visual | 0.001 | 1.71 | 0.09 | -0.0008 | -1.29 | 0.20 | -0.0009 | -1.76 | 0.09 |
| Bold indicates significant effects with p-value < .05, uncorrected. | | | | | | | | | |

| **Table S7:** Average Controllability Effect Estimates for the Go/No-Go Task. | | | | | | | | | |
| --- | --- | --- | --- | --- | --- | --- | --- | --- | --- |
|  | ADHD_Placebo_ vs. TD | | | ADHD_MPH_ vs. TD | | | ADHD_Placebo_ vs. ADHD_MPH_ | | |
| **Network** | **Estimate** | **t value** | **Pr(>\|t\|)** | **Estimate** | **t value** | **Pr(>\|t\|)** | **Estimate** | **t value** | **Pr(>\|t\|)** |
| Auditory | 0.0008 | 1.74 | 0.09 | -0.0004 | -0.80 | 0.43 | -0.0006 | -1.50 | 0.14 |
| Cingulo-Opercular | 0.0004 | 0.79 | 0.44 | 0.0003 | 0.72 | 0.48 | 0 | -0.04 | 0.97 |
| Default Mode | **0.0008** | **2.39** | **0.02** | 0 | -0.12 | 0.90 | -0.0004 | -1.46 | 0.15 |
| Dorsal Attention | **0.0011** | **2.36** | **0.02** | -0.0001 | -0.24 | 0.81 | -0.0006 | -1.48 | 0.15 |
| Fronto-Parietal | 0.0006 | 1.67 | 0.10 | 0.0001 | 0.16 | 0.87 | -0.0003 | -0.88 | 0.39 |
| Medial Temporal | **0.0016** | **2.07** | **0.04** | 0.0002 | 0.31 | 0.75 | -0.0007 | -0.97 | 0.34 |
| Parietal Memory | 0.0005 | 0.69 | 0.50 | -0.0006 | -0.70 | 0.48 | -0.0005 | -0.83 | 0.41 |
| Reward | **0.0018** | **2.80** | **0.007** | -0.001 | -1.52 | 0.13 | **-0.0014** | **-2.41** | **0.02** |
| Salience | 0.0005 | 0.97 | 0.34 | 0.0004 | 0.79 | 0.43 | 0 | -0.10 | 0.92 |
| Somatomotor Dorsal | **0.0009** | **2.35** | **0.02** | 0 | -0.13 | 0.90 | -0.0005 | -1.40 | 0.17 |
| Somatomotor Ventral | -0.0007 | -0.52 | 0.60 | -0.0004 | -0.32 | 0.75 | 0.0001 | 0.11 | 0.91 |
| Ventral Attention | 0.0004 | 0.76 | 0.45 | 0.0002 | 0.48 | 0.63 | -0.0001 | -0.15 | 0.88 |
| Visual | **0.0009** | **2.23** | **0.03** | -0.0006 | -1.59 | 0.12 | **-0.0008** | **-2.11** | **0.04** |
| Bold indicates significant effects with p-value < .05, uncorrected. | | | | | | | | | |

| **Table S9:** Modal Controllability Effect Estimates for the Resting State. | | | | | | | | | |
| --- | --- | --- | --- | --- | --- | --- | --- | --- | --- |
|  | ADHD_Placebo_ vs. TD | | | ADHD_MPH_ vs. TD | | | ADHD_Placebo_ vs. ADHD_MPH_ | | |
| **Network** | **Estimate** | **t value** | **Pr(>\|t\|)** | **Estimate** | **t value** | **Pr(>\|t\|)** | **Estimate** | **t value** | **Pr(>\|t\|)** |
| Auditory | 0.0002 | 0.41 | 0.69 | -0.0001 | -0.29 | 0.78 | -0.0001 | -0.38 | 0.70 |
| Cingulo-Opercular | -0.0003 | -1.07 | 0.29 | -0.0003 | -1.11 | 0.27 | 0 | -0.05 | 0.96 |
| Default Mode | 0.0002 | 0.55 | 0.58 | -0.0005 | -1.85 | 0.07 | -0.0003 | -1.36 | 0.18 |
| Dorsal Attention | 0.0004 | 0.97 | 0.34 | **-0.0011** | **-2.41** | **0.02** | -0.0007 | -1.91 | 0.06 |
| Fronto-Parietal | 0 | -0.03 | 0.98 | -0.0003 | -0.88 | 0.38 | -0.0001 | -0.49 | 0.63 |
| Medial Temporal | 0.0002 | 0.23 | 0.82 | -0.0006 | -0.79 | 0.43 | -0.0004 | -0.56 | 0.58 |
| Parietal Memory | 0.001 | 1.64 | 0.11 | -0.0001 | -0.21 | 0.84 | -0.0005 | -1.07 | 0.29 |
| Reward | -0.0002 | -0.41 | 0.68 | -0.0002 | -0.48 | 0.63 | 0 | -0.05 | 0.96 |
| Salience | 0.0001 | 0.30 | 0.77 | -0.0004 | -1.18 | 0.24 | -0.0002 | -0.83 | 0.41 |
| Somatomotor Dorsal | **-0.0006** | **-2.52** | **0.01** | 0.0003 | 1.09 | 0.28 | **0.0004** | **2.08** | **0.04** |
| Somatomotor Ventral | 0.0003 | 0.32 | 0.75 | 0.0016 | 1.62 | 0.11 | 0.0006 | 0.76 | 0.45 |
| Ventral Attention | 0.0006 | 1.55 | 0.13 | -0.0003 | -0.69 | 0.49 | -0.0004 | -1.26 | 0.21 |
| Visual | -0.0004 | -1.32 | 0.19 | 0.0003 | 0.92 | 0.36 | 0.0003 | 1.25 | 0.22 |
| Bold indicates significant effects with p-value < .05, uncorrected. | | | | | | | | | |

| **Table S10:** Modal Controllability Effect Estimates for the Go/No-Go Task. | | | | | | | | | |
| --- | --- | --- | --- | --- | --- | --- | --- | --- | --- |
|  | ADHD_Placebo_ vs. TD | | | ADHD_MPH_ vs. TD | | | ADHD_Placebo_ vs. ADHD_MPH_ | | |
| **Network** | **Estimate** | **t value** | **Pr(>\|t\|)** | **Estimate** | **t value** | **Pr(>\|t\|)** | **Estimate** | **t value** | **Pr(>\|t\|)** |
| Auditory | -0.0007 | -1.64 | 0.11 | 0.0003 | 0.74 | 0.46 | 0.0005 | 1.40 | 0.17 |
| Cingulo-Opercular | -0.0003 | -0.74 | 0.46 | -0.0003 | -0.77 | 0.44 | 0 | -0.02 | 0.99 |
| Default Mode | **-0.0006** | **-2.38** | **0.02** | 0 | 0.05 | 0.96 | 0.0003 | 1.42 | 0.16 |
| Dorsal Attention | **-0.0009** | **-2.29** | **0.03** | 0.0001 | 0.22 | 0.83 | 0.0005 | 1.43 | 0.16 |
| Fronto-Parietal | -0.0005 | -1.76 | 0.08 | 0 | -0.10 | 0.93 | 0.0002 | 0.98 | 0.33 |
| Medial Temporal | -0.0013 | -1.93 | 0.06 | -0.0003 | -0.41 | 0.69 | 0.0005 | 0.84 | 0.41 |
| Parietal Memory | -0.0004 | -0.64 | 0.52 | 0.0005 | 0.69 | 0.49 | 0.0004 | 0.79 | 0.43 |
| Reward | **-0.0015** | **-2.79** | **0.007** | 0.0008 | 1.48 | 0.15 | **0.0012** | **2.38** | **0.02** |
| Salience | -0.0004 | -0.93 | 0.36 | -0.0004 | -0.86 | 0.39 | 0 | 0.04 | 0.97 |
| Somatomotor Dorsal | **-0.0007** | **-2.27** | **0.03** | 0 | 0.05 | 0.96 | 0.0004 | 1.32 | 0.19 |
| Somatomotor Ventral | 0.0006 | 0.56 | 0.58 | 0.0003 | 0.33 | 0.74 | -0.0001 | -0.13 | 0.90 |
| Ventral Attention | -0.0003 | -0.75 | 0.46 | -0.0002 | -0.50 | 0.62 | 0.0001 | 0.13 | 0.90 |
| Visual | **-0.0007** | **-2.20** | **0.03** | 0.0005 | 1.57 | 0.12 | **0.0006** | **2.10** | **0.04** |
| Bold indicates significant effects with p-value < .05, uncorrected. | | | | | | | | | |

| **Table S11:** Modal Controllability Effect Estimates for the Rewarded Go/No-Go Task. | | | | | | | | | |
| --- | --- | --- | --- | --- | --- | --- | --- | --- | --- |
|  | ADHD_Placebo_ vs. TD | | | ADHD_MPH_ vs. TD | | | ADHD_Placebo_ vs. ADHD_MPH_ | | |
| **Network** | **Estimate** | **t value** | **Pr(>\|t\|)** | **Estimate** | **t value** | **Pr(>\|t\|)** | **Estimate** | **t value** | **Pr(>\|t\|)** |
| Auditory | -0.0006 | -0.91 | 0.36 | 1.00E-04 | 0.09 | 0.93 | 0.0003 | 0.57 | 0.57 |
| Cingulo-Opercular | -0.0006 | -1.14 | 0.26 | -0.0002 | -0.35 | 0.73 | 0.0002 | 0.44 | 0.66 |
| Default Mode | -0.0006 | -1.61 | 0.11 | 0.0001 | 0.32 | 0.75 | 0.0004 | 1.12 | 0.27 |
| Dorsal Attention | -0.001 | -1.84 | 0.07 | 0.0004 | 0.75 | 0.46 | 0.0007 | 1.53 | 0.13 |
| Fronto-Parietal | -0.0004 | -1.02 | 0.31 | 0.0002 | 0.46 | 0.65 | 0.0003 | 0.82 | 0.42 |
| Medial Temporal | -0.0015 | -1.52 | 0.13 | 0.0004 | 0.47 | 0.64 | 0.001 | 1.10 | 0.28 |
| Parietal Memory | -0.0013 | -1.82 | 0.07 | 0.0009 | 1.35 | 0.18 | **0.0011** | **2.06** | **0.046** |
| Reward | -0.0011 | -1.48 | 0.15 | 0.0008 | 1.10 | 0.28 | 0.0009 | 1.41 | 0.17 |
| Salience | -0.0001 | -0.21 | 0.84 | 0 | 0.05 | 0.96 | 0.0001 | 0.14 | 0.89 |
| Somatomotor Dorsal | **-0.0013** | **-2.40** | **0.02** | 0.0008 | 1.39 | 0.17 | **0.0011** | **2.18** | **0.04** |
| Somatomotor Ventral | **0.0027** | **2.91** | **0.005** | -0.0015 | -1.65 | 0.10 | **-0.0021** | **-2.93** | **0.005** |
| Ventral Attention | -0.0005 | -0.73 | 0.47 | 0.0005 | 0.74 | 0.47 | 0.0005 | 0.81 | 0.43 |
| Visual | -0.0009 | -1.74 | 0.09 | 0.0006 | 1.29 | 0.20 | 0.0008 | 1.79 | 0.08 |
| Bold indicates significant effects with p-value < .05, uncorrected. | | | | | | | | | |

**Analysis of Overlapping ADHD Subset**

This section reports the results of the supplemental analyses described in the *Participants* section of the *Main Text*. These analyses are identical to the analyses conducted in the *Main Text*, except that instead of defining MPH responders separately for each scan context, the subset of 13 children with ADHD who were classified as MPH responders for all scan contexts are included (Table S12). Briefly, two sets of mixed effects models were conducted for each of the three scan contexts (rest, go/no-go, rewarded go/no-go): (1) differences in network-level average and modal controllability between TD children and children with ADHD (both on MPH and on placebo); and (2) the effects of MPH on average and modal controllability in children with ADHD (on-off MPH). All models controlled for age, biological sex, and in-scanner motion (average framewise displacement without notch filtering, before censoring and processing), and used a random intercept at the level of the participant to account for participant-specific differences. All models were fit separately to each network, resulting in 156 models in total. See Figures S1 and S2 for a summary of the findings in this subset of participants with ADHD.

**Table S12:** Sample Characteristics for Overlapping ADHD Subset.

|  | **ADHD (*n* = 13)** | **TD (*n* = 27)** |
| --- | --- | --- |
| Age (years) | 10.06 (1.21) | 10.38 (1.45) |
| # Female | 6 (46.1%) | 11 (40.7%) |
| Framewise Displacement (mm) | 0.150 (0.077) | 0.146 (0.075) |
| ADHD Symptoms^a^ |  |  |
| Inattentive | 1.89 (0.74) | 0.26 (0.39) |
| Hyperactive/Impulsive | 1.42 (0.72) | 0.12 (0.14) |

All results are presented as mean (SD) or number (%). ADHD = attention-deficit/hyperactivity disorder. TD = typically developing.

^a^ ADHD symptoms were assessed using the *Swanson, Nolan, and Pelham Rating Scale, Version IV* (SNAP-IV) ^3^*.*


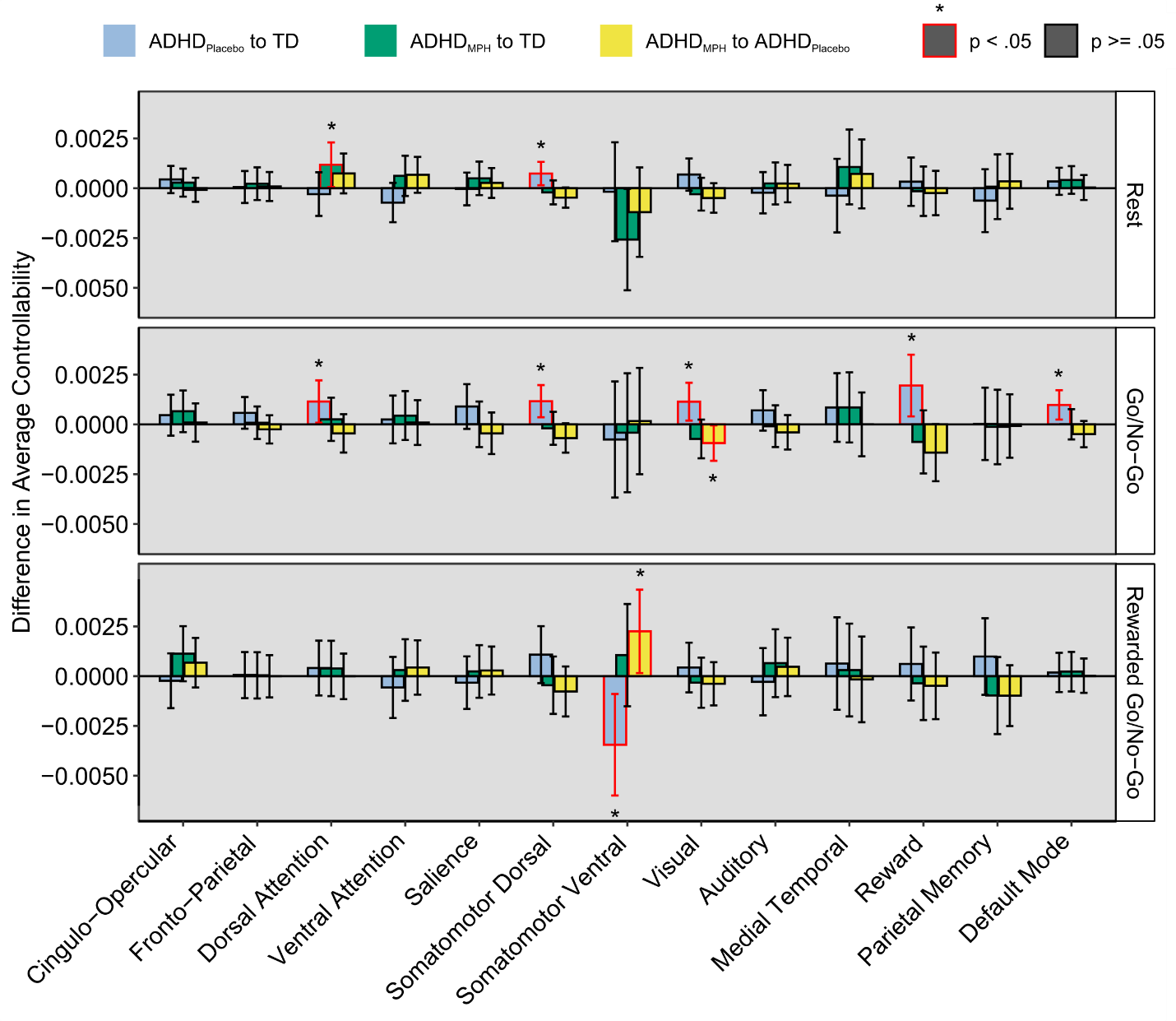


**Figure S1:** Average controllability in TD subjects and a subset of subjects with ADHD who were classified as MPH responders for all tasks (n = 13). The figure presents the difference in average controllability between ADHD on placebo and TD (blue bars), ADHD on MPH and TD (green bars), and within ADHD (yellow bars) for the three functional scan contexts (rest, go/no-go, rewarded go/no-go). Children with ADHD on placebo exhibited increased average controllability in the somatomotor dorsal network during rest, and the dorsal attention, somatomotor dorsal, visual, reward, and default mode networks during the go/no-go task. Children with ADHD on placebo exhibited decreased average controllability in the somatomotor ventral network during the rewarded go/no-go task. During rest, children with ADHD on MPH exhibited higher average controllability in the dorsal attention network. For all cases in which children with ADHD on placebo exhibited significantly different average controllability compared to TD children, those differences were no longer significant when comparing children with ADHD on MPH and TD children. This was supported by significant within-ADHD effects of MPH for the visual network during the go/no-go task and the somatomotor ventral network during the rewarded go no-go task. Results are highly consistent with those reported in the *Main Text*, with the following differences in terms of significance levels: (1) children with ADHD no longer show significant reductions in average controllability on MPH as compared to on placebo of the somatomotor dorsal network during rest or of the reward network during the go/no-go task; and (2) there are no longer significant differences between children with ADHD on placebo and TD children, or between children with ADHD on MPH and on placebo, in the somatomotor dorsal network during the rewarded go/no-go task. Critically, magnitude and direction of effects remained consistent in this restricted sample as compared to results reported in the *Main Text*. Bars correspond to the regression estimate of the relevant difference, controlling for age, biological sex, and in-scanner motion. Error bars correspond to 95% confidence intervals. Red outline and asterisk indicate statistical significance at p < .05.


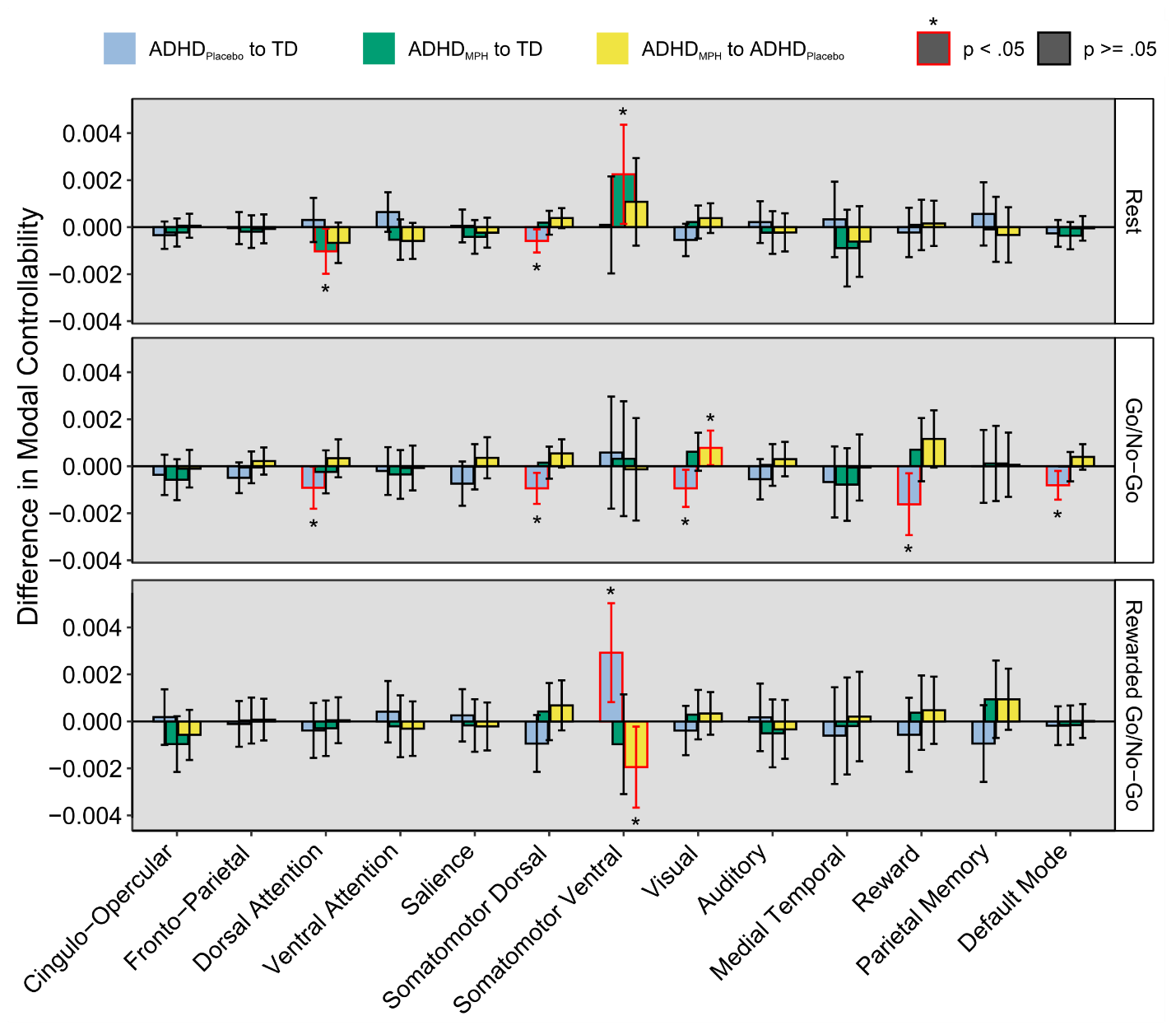
**Figure S2:** Modal controllability in TD subjects and a subset of subjects with ADHD who were classified as MPH responders for all tasks (n = 13). The figure presents the difference in modal controllability between ADHD on placebo and TD (blue bars), ADHD on MPH and TD (green bars), and within ADHD (yellow bars) for the three functional scan contexts (rest, go/no-go, rewarded go/no-go). Children with ADHD on placebo exhibited decreased modal controllability in the somatomotor dorsal network during rest, and in the dorsal attention, somatomotor dorsal, visual, reward, and default mode networks during the go/no-go task. Children with ADHD on placebo exhibited increased modal controllability in the somatomotor ventral network during the rewarded go/no-go task. During rest, children with ADHD on MPH exhibited lower modal controllability in the dorsal attention network and higher modal controllability in the somatomotor ventral network. For all cases in which children with ADHD on placebo exhibited significantly different modal controllability compared to TD children, those differences were no longer significant when comparing children with ADHD on MPH and TD children. This was supported by significant within-ADHD effects of MPH for the visual network during the go/no-go task and the somatomotor dorsal network during the rewarded go no-go task. Results are highly consistent with those reported in the *Main Text*, with the following differences in terms of significance levels: (1) children with ADHD on MPH show higher modal controllability than TD children in the somatomotor ventral network during rest; (2) children with ADHD no longer show significant increases in modal controllability on MPH as compared to on placebo of the somatomotor dorsal network during rest or of the reward network during the go/no-go task; and (3) there are no longer significant differences between children with ADHD on placebo and TD children, or between children with ADHD on MPH and on placebo, in the somatomotor dorsal network during the rewarded go/no-go task. Critically, magnitude and direction of effects remained consistent in this restricted sample as compared to results reported in the *Main Text*. Bars correspond to the regression estimate of the relevant difference, controlling for age, biological sex, and in-scanner motion. Error bars correspond to 95% confidence intervals. Red outline and asterisk indicate statistical significance at p < .05.

**References**

1 Shaffer D, Fisher P, Lucas CP, Dulcan MK, Schwab-Stone ME. NIMH Diagnostic Interview Schedule for Children Version IV (NIMH DISC-IV): Description, differences from previous versions, and reliability of some common diagnoses. *J Am Acad Child Adolesc Psychiatry* 2000; **39**: 28–38.

2 Conners CK. *Conners 3*. 3rd ed. Pearson: London, 2008.

3 Swanson JM. *SNAP-IV Scale*. University of California Child Development Center: Irvine, CA, 1995.

4 Wechsler D. *WISC​-V: Technical and Interpretive Manual*. Pearson: Bloomington, MN, 2014.

5 Wechsler D. *Wechsler Individual Achievement Test*. 3rd ed. The Psychological Corporation: Bloomington, MN, 2009.

6 Esteban O, Markiewicz CJ, Blair RW, Moodie CA, Isik AI, Erramuzpe A *et al.* fMRIPrep: A robust preprocessing pipeline for functional MRI. *Nat Methods* 2019; **16**: 111–116.

7 Esteban O, Markiewicz CJ, Goncalves M, DuPre E, Kent JD, Salo T *et al.* *fMRIPrep: A robust preprocessing pipeline for functional MRI*. Zenodo, 2020 doi:10.5281/ZENODO.852659.

8 Gorgolewski K, Burns CD, Madison C, Clark D, Halchenko YO, Waskom ML *et al.* Nipype: A flexible, lightweight and extensible neuroimaging data processing framework in Python. *Front Neuroinformatics* 2011; **5**: 13.

9 Esteban O, Markiewicz CJ, Burns C, Goncalves M, Jarecka D, Ziegler E *et al.* *nipy/nipype: 1.5.1*. Zenodo, 2020 doi:10.5281/ZENODO.596855.

10 Tustison NJ, Avants BB, Cook PA, Zheng Y, Egan A, Yushkevich PA *et al.* N4ITK: Improved N3 bias correction. *IEEE Trans Med Imaging* 2010; **29**: 1310–1320.

11 Avants BB, Epstein CL, Grossman M, Gee JC. Symmetric diffeomorphic image registration with cross-correlation: Evaluating automated labeling of elderly and neurodegenerative brain. *Med Image Anal* 2008; **12**: 26–41.

12 Zhang Y, Brady M, Smith S. Segmentation of brain MR images through a hidden Markov random field model and the expectation-maximization algorithm. *IEEE Trans Med Imaging* 2001; **20**: 45–57.

13 Reuter M, Rosas HD, Fischl B. Highly accurate inverse consistent registration: A robust approach. *NeuroImage* 2010; **53**: 1181–1196.

14 Dale AM, Fischl B, Sereno MI. Cortical surface-based analysis: I. Segmentation and surface reconstruction. *NeuroImage* 1999; **9**: 179–194.

15 Klein A, Ghosh SS, Bao FS, Giard J, Häme Y, Stavsky E *et al.* Mindboggling morphometry of human brains. *PLoS Comput Biol* 2017; **13**: e1005350.

16 Fonov V, Evans A, McKinstry R, Almli C, Collins D. Unbiased nonlinear average age-appropriate brain templates from birth to adulthood. *NeuroImage* 2009; **47**: S102–S102.

17 Wang S, Peterson DJ, Gatenby JC, Li W, Grabowski TJ, Madhyastha TM. Evaluation of field map and nonlinear registration methods for correction of susceptibility artifacts in diffusion MRI. *Front Neuroinformatics* 2017; **11**: 17.

18 Huntenburg JM, Gorgolewski KJ, Anwander A, Margulies DS. Evaluating nonlinear coregistration of BOLD EPI and T1w images. *F1000Research* 2014; **5**.

19 Treiber JM, White NS, Steed TC, Bartsch H, Holland D, Farid N *et al.* Characterization and correction of geometric distortions in 814 diffusion weighted images. *PLOS ONE* 2016; **11**: e0152472.

20 Greve DN, Fischl B. Accurate and robust brain image alignment using boundary-based registration. *NeuroImage* 2009; **48**: 63–72.

21 Jenkinson M, Bannister P, Brady M, Smith S. Improved optimization for the robust and accurate linear registration and motion correction of brain images. *NeuroImage* 2002; **17**: 825–841.

22 Cox RW, Hyde JS. Software tools for analysis and visualization of fMRI data. *NMR Biomed* 1997; **10**: 171–178.

23 Power JD, Mitra A, Laumann TO, Snyder AZ, Schlaggar BL, Petersen SE. Methods to detect, characterize, and remove motion artifact in resting state fMRI. *NeuroImage* 2014; **84**: 320–341.

24 Satterthwaite TD, Elliott MA, Gerraty RT, Ruparel K, Loughead J, Calkins ME *et al.* An improved framework for confound regression and filtering for control of motion artifact in the preprocessing of resting-state functional connectivity data. *NeuroImage* 2013; **64**: 240–256.

25 Lanczos C. Evaluation of noisy data. *J Soc Ind Appl Math Ser B Numer Anal* 1964; **1**: 76–85.

26 Abraham A, Pedregosa F, Eickenberg M, Gervais P, Mueller A, Kossaifi J *et al.* Machine learning for neuroimaging with scikit-learn. *Front Neuroinformatics* 2014; **8**: 14.

27 Seitzman BA, Gratton C, Marek S, Raut RV, Dosenbach NUF, Schlaggar BL *et al.* A set of functionally-defined brain regions with improved representation of the subcortex and cerebellum. *NeuroImage* 2020; **206**: 116290.

28 Nebel MB, Lidstone DE, Wang L, Benkeser D, Mostofsky SH, Risk BB. Accounting for motion in resting-state fMRI: What part of the spectrum are we characterizing in autism spectrum disorder? *NeuroImage* 2022; **257**: 119296.
